# False-Positive Malaria Rapid Diagnostic Test Likely Due to African Tick Bite Fever: A Case Report

**DOI:** 10.3390/reports7040100

**Published:** 2024-11-16

**Authors:** Rahel T. Zewude, Syed Zain Ahmad, Tom Joseph, Andrea K. Boggild

**Affiliations:** 1 Division of Infectious Diseases, Department of Medicine, University of Toronto, Toronto, ON M5S 3H2, Canada; 2 Department of Laboratory Medicine and Pathobiology, University of Toronto, Toronto, ON M5T 3M7, Canada; 3 Institute of Medical Science, University of Toronto, Toronto, ON M5T 3M7, Canada; 4 Lawrence Bloomberg Faculty of Nursing, University of Toronto, Toronto, ON M5T 3M7, Canada; 5 Tropical Disease Unit, Toronto General Hospital, Toronto, ON M5G 2C4, Canada

**Keywords:** fever in the returning traveler, malaria, rapid diagnostic test, rickettsioses, sensitivity, specificity

## Abstract

**Background and Clinical Significance**: Fever in the returning traveler is a medical emergency warranting prompt exclusion of potentially life-threatening infections such as malaria. **Case Presentation**: We describe a case of a febrile returned traveler to South Africa whose prompt initial diagnostic work-up was notable for a false-positive malaria rapid diagnostic test (RDT), and who nevertheless responded quickly to oral atovaquone-proguanil, despite an ultimate diagnosis of African tick bite fever. Subsequent RDT and malaria thick- and thin-film blood examination failed to corroborate a diagnosis of malaria and all other microbiological testing other than rickettsial serology remained non-contributory. **Conclusions**: The case presented highlights important points regarding diagnostic test performance characteristics and premature diagnostic closure.

## 1. Introduction and Clinical Significance

Fever in the returning traveler is a common clinical scenario encountered in emergency and outpatient medicine and warrants immediate exclusion of life-threatening infections such as malaria, bacteremia, and meningitis. Malaria is caused by protozoa belonging to the genus *Plasmodium*, typically transmitted to humans via *Anopheles* mosquitoes. Of the five main *Plasmodium* species known to infect humans, *Plasmodium falciparum* is the species associated with the highest rates of mortality. The disease occurs primarily in tropical and subtropical regions of Africa, Central and South America, Asia, and Oceania. While the morbidity and mortality caused by the disease has declined globally since 2000 due to increased malaria control interventions, as of 2021 there were still an estimated 247 million cases of malaria worldwide, with 619,000 deaths. Africa, where over 90% of all malaria deaths occur, continues to disproportionately carry the highest share of the global disease burden [1,2].

Standard of care requires that exclusion of malaria occurs within 2 h of presentation to health care, and as such, rapid, antigen-based point-of-care and laboratory tests are widely available and used. Such rapid diagnostic tests (RDTs) are highly sensitive for the deadliest form of malaria, *Plasmodium falciparum*, and as such are a cost-effective and life-saving diagnostic intervention. However, as with all microbiological assays, performance limitations include rare instances of false positivity and false negativity.

We herein describe a case of a febrile returned traveler to South Africa whose initial diagnostic work-up led to a false diagnosis of malaria. In South Africa, malaria prevalence is geographically restricted, but regions near the borders with Mozambique, Zimbabwe, and Eswatini continue to have higher transmission rates, including the Limpopo Province [3], which our patient visited. Clinically, one of the major risks of false-positive malaria rapid testing is premature diagnostic closure in the febrile returned traveler, followed by the initiation of ineffective therapy. Needless administration of anti-malarials can not only harm the patient, through side effects and adverse events, but can also contribute to anti-malarial resistance through overprescription. Exclusion of potentially life-threatening illnesses such as bacteremia is still required in the febrile returned traveler with or without positive malaria testing results, given the possibility of coinfection and rapid clinical progression [4,5].

## 2. Case Presentations

A 58-year-old man presented to the emergency department with a 5-day history of fevers, chills, arthralgias, and myalgias that began a day after he returned to Toronto, Canada, from a work trip to South Africa. His past medical history was notable for hypertension, dyslipidemia, and diabetes, for which he was taking standard first-line therapeutics.

Travel history was notable for a 10-day trip to South Africa in late May, where he stayed in Johannesburg with shared accommodation with colleagues. During his time in South Africa, the patient had taken day trips to visit mining sites in high-rainfall regions in the Limpopo province. Prior to this visit, he had traveled to South Africa every month for similar work trips.

Over one year prior to this trip, he had received vaccination for yellow fever, typhoid, hepatitis A and B, as well as a tetanus booster. He did not use any malaria prophylaxis during this trip. He did not use insect repellents or other arthropod personal protective measures during this trip.

Notable exposures during this trip included approximately 15 arthropod bites that occurred on his right arm, bilateral hips, ankles, and wrist. He described them as small red bite wounds with no ulcers or dark lesions. His small bite wounds had no associated discharge or pain, but he noted mild pruritus. These bite wounds had already started to improve spontaneously over the few days following each bite.

Notably, two other colleagues who stayed at the shared accommodation and visited the same mining sites also had experienced similar arthropod bites and developed similar fevers, chills, arthralgias, and myalgias upon their return from the work trip.

He could not identify the type of arthropod that bit him and he could not recall any mosquito bites. Otherwise, he had no animal exposures during this visit.

His symptoms of fevers, chills, myalgia, and arthralgia began 1 day after his return to Canada. Upon presentation for pre-consultation blood work, healing arthropod bites and eschars of the right arm, ankles, and wrists were noted. His initial blood work demonstrated a normal hemoglobin of 142 g/L [normal: 120–160 g/L], platelets of 246 × 10^9^/L [normal: 150–400 × 10^9^/L], and white blood cell count of 6.2 × 10^9^/L [normal: 4–11 × 10^9^/L]. His creatinine was 72 µmol/L [normal 49–93 µmol/L], and aspartate aminotransferase (AST) and alanine aminotransferase (ALT) were mildly elevated at 60 IU/L [normal: 15–37 IU/L] and 94 IU/L [normal: 17–63 IU/L], respectively, while total bilirubin was normal at 6 µmol/L [normal 3–17 µmol/L].

Microbiological investigations were notable for a malaria rapid diagnostic test (RDT) (BinaxNOW^®^, Alere, Stittsville, ON, USA) that was positive for detection of both *P. falciparum*-specific histidine-rich protein II (HRP-2) antigen (T1 band) and pan-malarial aldolase antigen (T2 band). Following the positive RDT result at the hospital, his whole blood specimen was sent to the provincial reference parasitology laboratory for confirmatory testing. RDT (BinaxNOW^®^, Alere) at the reference laboratory returned negative. Thick- and thin-film microscopy were reviewed by a single senior Medical Laboratory Technologist with over 5 years of parasitology experience, and no parasitized erythrocytes were visualized on three separate occasions during acute illness. Based on the reference laboratory’s diagnostic algorithm, no malaria polymerase chain reaction (PCR) was conducted as both reference RDT and smears had resulted as negative. Two sets of blood cultures were drawn and remained negative for growth of routine, non-fastidious aerobic and anaerobic bacteria.

Following the positive RDT result at the hospital, but before final reporting of the reference laboratory testing results, the patient was prescribed atovaquone–proguanil 250–100 mg, 4 tablets daily with food for 3 days, which he completed. He was then evaluated in our unit and given his negative thick and thin smears, and travel history to rural South African with arthropod bite exposure, a clinical diagnosis of African tick bite fever (ATBF) was suggested. As such, rickettsial serologies through indirect immunofluorescent assay (IFA) were arranged. IFA to detect IgG antibodies to rocky mountain spotted fever (RMSF) group which included *Rickettsia rickettsii*, *Rickettsia akari,* and *Rickettsia africae* antigens, as well as IFA to detect IgG antibodies to typhus fever group, which included *Rickettsia typhi* and *Rickettsia prowazekii*, were conducted. Repeat malaria RDT along with thin and thick smears were also conducted. As the patient was clinically well by this point, no antibiotic therapy was prescribed. His RMSF group IgG serology returned reactive at a titre of 1:1024. Conversely, his typhus fever group IgG serology returned non-reactive with a titre of <1:64.

Final malaria testing was conducted on day 3 of his atovaquone–proguanil course, and his RDT (BinaxNOW^®^, Alere, Stittsville, ON, USA) returned negative for both *P. falciparum*-specific HRP2 antigen and pan-*Plasmodium* aldolase. Blood microscopy remained negative.

Given both clinical and serological evidence of ATBF and an absence of parasitologic confirmation of malaria, the initial positive RDT was thought to represent a false positive result.

His fevers, chills, arthralgia, and myalgia symptoms had resolved by his second day of atovaquone–proguanil which occurred 13 days after his symptom onset. His bite wounds also had scabbed over by this time with small eschars present in the middle of each red bite wound. He developed no further lesions post-travel, again, supporting the diagnosis of ATBF rather than other common dermatoses such as furunculosis or secondarily infected bite wounds. The patient convalesced well from his ATBF and required no further antimicrobials. At his next follow-up assessment, 3 months after his return from the trip, he had completely recovered with resolution of all bite lesions and eschars.

## 3. Discussion

Malaria is one of the most common, specific etiologic causes of fever in travelers to the tropics and warrants prompt exclusion in order to avert death and morbidity. Traditionally, malaria is diagnosed via Giemsa-stained microscopy of thick and thin smear preparations, which allow for species identification, quantitative assessment of parasitemia, specification of life cycle stages (i.e., ring-stage trophozoites, schizonts, and gametocytes, notably) as well as monitoring of treatment response. However, microscopy requires labor-intensive specimen processing and skilled microscopists. Due to their mass distribution and ease of use, RDTs have emerged as an alternative method for rapid diagnosis and most notably exclusion of *P. falciparum* infection.

RDTs are lateral flow devices which employ immunochromatographic assays that use monoclonal antibodies to detect specific antigens associated with *Plasmodium* spp. in a blood sample, producing a visible color change [6]. The antigens they commonly detect are histidine-rich protein 2 (HRP-2), which is a specific target for *P. falciparum*, and pan-*Plasmodium* spp. antigens such as aldolase or *Plasmodium* lactate dehydrogenase (pLDH) [7]. RDTs that solely detect the HRP-2 antigen are generally less costly but as research has demonstrated they have variable sensitivity across regions of the world due to genetic variability in deletions of HRP-2 [7].

In our patient’s case, the BinaxNOW^®^ RDT was used, which offered a significant improvement in the sensitivity of malaria detection through its ability to qualitatively detect both HRP-2 and aldolase, a protein which is present in all malaria species [8].

A 2012 study by DiMaio and colleagues assessing the performance of BinaxNOW for the diagnosis of malaria in a US hospital determined that the RDT was 84.2% sensitive for patients on antimalarial therapy and 92.9% sensitive for those who were not and 99.8% specific [9]. Importantly, the authors reported that BinaxNOW^®^ also misclassified a case of *P. falciparum* infection as non-*falciparum* [9]. Another study by Lee and colleagues sought to evaluate four different RDTs for malaria by testing healthy control patients, *P. vivax*-infected patients, and patients with positive rheumatoid factor but no malaria [10]. BinaxNOW^®^ was found to have the highest false-positive rate by specimen, at 13% with a rate of 9.8% for the HRP-2 band and 5.4% for the aldolase band [10]. In recent years, there have been several reports of false-positive BinaxNOW^®^ results associated with chronic hepatitis C, toxoplasmosis, dengue, human African trypanosomiasis, leishmaniasis, Chagas disease, schistosomiasis, heterophile antibodies, and, most commonly, rheumatoid factor [11]. Accordingly, it is possible that our patient was not only suffering from ATBF but had also been exposed to another microbial entity—particularly dengue or mononucleosis—that could have been responsible for the RDT false positivity.

To the best of our knowledge, there have not been any prior reports of false positive BinaxNOW^®^ RDTs due to ATBF. ATBF is caused by the intracellular bacteria, *Rickettsia africae*, first isolated in 1992 by Kelly and colleagues, that is transmitted by the *Amblyomma* species ticks found mainly in the regions of South Africa and sub-Saharan Africa [12]. Unlike other ticks that passively wait for hosts on vegetation, the *Amblyomma* ticks of ATBF, *A. variegatum* and *A. hebraeum*, actively move towards their hosts, primarily cattle [13]. As such, multi-eschar disease in ATBF is common. Once an infected tick feeds on a human, the incubation period for *R. africae* is usually 5 to 10 days [13]. Patients often present with an eschar at the site of inoculation, and with clusters of eschars if multiple bites have occurred, as is often the case with *Amblyomma* species [14]. Other common presenting symptoms include fever, localized tender lymphadenitis, maculopapular rash, myalgias, and headaches [13,14].

While ATBF is typically a clinical diagnosis, with limited availability of rapid laboratory diagnostics, a microbiological diagnosis can be established serologically, via immunohistochemistry that uses monoclonal antibodies for *R. africae* antigen detection or immunofluorescence assay (IFA) which uses fluorescein-labeled antibody [12,15].

The use of polymerase chain reaction (PCR), the primers of which target specific *Rickettsiales* sequences, on eschar biopsies, has also been described in reported cases and research studies [12,16]. However, PCR is not a routinely used diagnostic modality given challenges of obtaining skin biopsies and limited laboratory experience and resources to process rickettsia PCR [12,15]. Similarly, culture is a resource-intensive method that is restricted to specialized reference laboratories, given the obligate intracellular nature of *R. africae* and its requirement for cell culture [13].

The clinical diagnosis of ATBF is supported by characteristic manifestations such as inoculation eschar, also known as tache noir, and highly suggestive findings such as regional lymphadenitis along with fever with compatible geographical area of infection [13]. Along with ATBF, the differential diagnosis of fever in a traveler with spotted rash includes both vector-borne infections such as dengue, Chikungunya, Zika, and relapsing fever, as well as non-vector-borne infections such as syphilis, meningococcemia, measles, varicella, HIV, and Coxsackievirus. Clinicians should therefore maintain a high degree of vigilance and a broad differential diagnosis with thorough diagnostic approach to patients presenting for care with such a clinical syndrome following tropical travel [5]. Following recommended clinical diagnostic algorithms, such as those presented by Thwaites and colleagues [5], ensures consideration and exclusion of potentially severe vector-borne and non-vector-borne travel-acquired infections that may be underdiagnosed and underreported due to lack of awareness on the part of clinical teams outside of endemic areas.

If there are reasonable clinical grounds to suspect ATBF, the patient remains unwell, and alternate diagnosis have been excluded either clinically or microbiologically, empiric treatment with doxycycline for 1-week is recommended [12,14]. Rapid recovery is often seen in patients after treatment with doxycycline but severe manifestations of myocarditis and subacute neuropathy have been reported in elderly patients [12,13].

Notably, laboratory abnormalities seen with ATBF typically include lymphopenia, transaminitis, and thrombocytopenia which can also occur with malaria [14].

A multi-centered GeoSentinel network study of over 17,000 ill returned travelers has identified ATBF to be the second most common etiology of fever for travelers from sub-Saharan Africa [16,17]. Therefore, it is prudent for clinicians to note that ATBF can be a potential cause of false positive malaria RDT. In patients with compatible travel history to Southern or sub-Saharan Africa, with multiple negative malaria thick and thin smears and skin lesions, particularly if any are tender, the diagnosis of ATBF should be considered, and prompt thorough examination for inoculation eschars should occur. Patients who are symptomatic at presentation who have received a clinical diagnosis of ATBF should be offered doxycycline therapy, or a macrolide in the event of contraindication to doxycycline.

## 4. Conclusions

The case reported herein illustrates the challenges encountered with rapid diagnostics in patients with acute tropical infectious diseases. While the risk of malaria in febrile returned travelers necessitates prompt exclusion of the diagnosis, the rapidity with which RDT results are rendered raises the concern for premature diagnostic closure and inadvertent treatment in those for whom initial diagnostic testing is falsely positive. At the same time, critical alternate diagnoses for which the turnaround time of microbiological confirmation may be prolonged—such as dengue fever, bacteremia, and acute rickettsioses—may not be pursued in ill returned travelers who have been diagnosed with malaria. Given the possibility of intercurrent infections as well as test performance limitations in the acutely unwell traveler, ensuring exclusion of all the most likely and life-threatening etiologies, as well as those with significant public health impact is of utmost importance.

## Data Availability

The original contributions presented in the study are included in the article, further inquiries can be directed to the corresponding author. The data are not publicly available due to privacy concerns.

## References

[1] World Health Organization (2023). Malaria. https://www.who.int/news-room/fact-sheets/detail/malaria.

[2] Walter K., John C.C. (2022). Malaria. JAMA.

[3] Raman J., Morris N., Frean J., Brooke B., Blumberg L., Kruger P., Mabusa A., Raswiswi E., Shandukani B., Misani E. (2016). Reviewing South Africa’s malaria elimination strategy (2012–2018): Progress, challenges and priorities. Malar. J..

[4] Boggild A., Ghesquiere D.W., McCarthy D.A., CATMAT (2011). Fever in the Returning International Traveller Initial Assessment Guidelines: Committee to Advise on Tropical Medicine and Travel (CATMAT). Can. Commun. Dis. Rep..

[5] Thwaites G.E., Day N.P. (2017). Approach to Fever in the Returning Traveler. N. Engl. J. Med..

[6] Kavanaugh M.J., Azzam S.E., Rockabrand D.M. (2021). Malaria Rapid Diagnostic Tests: Literary Review and Recommendation for a Quality Assurance, Quality Control Algorithm. Diagnostics.

[7] Mukkala A.N., Kwan J., Lau R., Harris D., Kain D., Boggild A.K. (2018). An Update on Malaria Rapid Diagnostic Tests. Curr. Infect. Dis. Rep..

[8] Phuong M., Lau R., Ralevski F., Boggild A.K. (2015). Survival analysis of diagnostic assays in *Plasmodium falciparum* malaria. Malar. J..

[9] Dimaio M.A., Pereira I.T., George T.I., Banaei N. (2012). Performance of BinaxNOW for diagnosis of malaria in a U.S. hospital. J Clin Microbiol.

[10] Lee J.H., Jang J.W., Cho C.H., Kim J.Y., Han E.T., Yun S.G., Lim C.S. (2014). False-positive results for rapid diagnostic tests for malaria in patients with rheumatoid factor. J. Clin. Microbiol..

[11] Haberichter K.L., Johnson P.C., Chittick P.J., Millward P., Robinson-Dunn B., Boyanton B.L. (2017). The brief case: False-positive rapid malaria antigen test result in a returned traveler. J. Clin. Microbiol..

[12] Parola P., Paddock C.D., Socolovschi C., Labruna M.B., Mediannikov O., Kernif T., Abdad M.Y. (2013). Update on tick-borne rickettsioses around the world: A geographic approach. Clin. Microbiol. Rev..

[13] Althaus F., Greub G., Raoult D., Genton B. (2010). African tick-bite fever: A new entity in the differential diagnosis of multiple eschars in travelers. Description of five cases imported from South Africa to Switzerland. Int. J. Infect. Dis..

[14] Daneman N., Slinger R. (2008). Tache noire. CMAJ.

[15] Jensenius M., Fournier P.E., Kelly P., Myrvang B., Raoult D. (2003). African tick bite fever. Lancet Infect. Dis..

[16] Nilsson K., Wallménius K., Rundlöf-Nygren P., Strömdahl S., Påhlson C. (2017). African tick bite fever in returning Swedish travellers. Report of two cases and aspects of diagnostics. Infect. Ecol. Epidemiol..

[17] Leder K., Torresi J., Libman M.D., Cramer J.P., Castelli F., Schlagenhauf P., Freedman D.O. (2013). GeoSentinel surveillance of illness in returned travelers, 2007–2011. Ann. Intern. Med..

